# The Effects of Tight-Lacing

**Published:** 1889-04

**Authors:** 


### THE EFFECTS OF TIGHT-LACING.

Dr. Boris I Kianovsky records, in some recent numbers of *Vratch*, a series of experiments that he has carried out with the view of determining the effect of tight-lacing on vital capacity, the movements of the thorax, the energy of inspiration and expiration, arterial tension, pulse, and respiration. The experiments were conducted on thirty female patients between the ages of 18 and 44 years, twenty-eight of whom were tight-lacers. His results may be summarized as follows:

1. The corset lessens the respiratory movement of the thorax, and diminishes both the vital capacity and the force of the inspirations and expirations, the inspiratory movements being particularly affected.

2. Since the corset compresses the thorax and diminishes the amount of the inspired air, it necessarily gives rise to chronic "oxygen starvation," which is one of the chief causes of dyspnœa and cardiac palpitation during brisk walking (fatigue being noticed early on physical or mental exertion), anorexia, faintness, vertigo, and other like symptoms usually noticed by tight-lacers.

3. A tight corset causes a fall of arterial tension, which in tight-lacers is usually below the normal (consequent upon arterial anæmia).

4. The effect of the corset on the frequency of the pulse was shown by the following experiment: The women were made to run 980 feet, with moderate swiftness, without corsets or lacing. The pulse was 136, 140, and 156, and the respiration 32 a minute. When the same women ran the distance tight-laced the pulse was 144, 160, and 176, and the respiration 48, 60, and 64.

Among thirty-eight corset-wearers, movable kidney was found in eight, habitual constipation or gastro-intestinal catarrh in fourteen, disease of the apex of the lungs in six, anæmia in five, hysteria in five.

The author says in conclusion: "I cannot help saying that I look upon the work of my predecessors and upon my own humble contribution with a sense of bitter and painful regret, for I am conscious that all the labor directed towards showing the evil effects of tight-lacing must yet remain unnoticed, or neglected, by women for a very long time." Nevertheless, such work is in the direction of desired dress reform, and if persisted in must in time produce some result.

